# Prone positioning improves oxygenation and lung recruitment in patients with SARS-CoV-2 acute respiratory distress syndrome; a single centre cohort study of 20 consecutive patients

**DOI:** 10.1186/s13104-020-05426-2

**Published:** 2021-01-09

**Authors:** Jennifer Clarke, Pierce Geoghegan, Natalie McEvoy, Maria Boylan, Orna Ní Choileáin, Martin Mulligan, Grace Hogan, Aoife Keogh, Oliver J. McElvaney, Oisín F. McElvaney, John Bourke, Bairbre McNicholas, John G. Laffey, Noel G. McElvaney, Gerard F. Curley

**Affiliations:** 1 Department of Anaesthesia and Critical Care, Royal College of Surgeons Ireland, Smurfit Building, Beaumont Hospital, Dublin 9, D09 YD60 Ireland; 2 Beaumont Hospital, Dublin 9, Ireland; 3 Galway University Hospital, University Road, Galway, Ireland

**Keywords:** Respiratory distress syndrome, Adult, Prone position, Severe acute respiratory syndrome coronavirus 2

## Abstract

**Objective:**

We aimed to characterize the effects of prone positioning on respiratory mechanics and oxygenation in invasively ventilated patients with SARS-CoV-2 ARDS.

**Results:**

This was a prospective cohort study in the Intensive Care Unit (ICU) of a tertiary referral centre. We included 20 consecutive, invasively ventilated patients with laboratory confirmed SARS-CoV-2 related ARDS who underwent prone positioning in ICU as part of their management. The main outcome was the effect of prone positioning on gas exchange and respiratory mechanics. There was a median improvement in the PaO_2_/FiO_2_ ratio of 132 in the prone position compared to the supine position (IQR 67–228). We observed lower PaO_2_/FiO_2_ ratios in those with low (< median) baseline respiratory system static compliance, compared to those with higher (> median) static compliance (*P* < 0.05). There was no significant difference in respiratory system static compliance with prone positioning. Prone positioning was effective in improving oxygenation in SARS-CoV-2 ARDS. Furthermore, poor respiratory system static compliance was common and was associated with disease severity. Improvements in oxygenation were partly due to lung recruitment. Prone positioning should be considered in patients with SARS-CoV-2 ARDS.

## Introduction

Acute Respiratory Distress Syndrome (ARDS) resulting from SARS-CoV-2 infection has a high mortality rate (> 40%) [1]. It has been demonstrated that prone positioning reduces mortality in non COVID-19 (“classic”) severe ARDS [2]. This may be due to optimized lung recruitment, reduced lung strain, and more homogeneous and therefore lung-protective ventilation in the prone position [3]. However, patients with COVID-19 pneumonia fulfilling the Berlin criteria for ARDS [4] may present with an atypical form of the syndrome [5–7]. In particular it has been suggested that the majority of patients with SARS-CoV-2 ARDS have relatively compliant lungs with low recruitability [5, 6]. This could imply that the response to prone positioning may differ in SARS-CoV-2 ARDS compared to “classic” ARDS. In particular, lung recruitment should not occur in the prone position in compliant lungs. This should result in (1) smaller improvements in oxygenation than seen in “classic” ARDS and (2) a reduction in total respiratory system compliance (because of the failure of lung recruitment to compensate for the reduction in chest wall compliance that is consistently seen in prone positioning [8]).

The response to prone positioning in SARS-CoV-2 ARDS has not been well described. We aimed to characterize this response. We hypothesized that poor compliance would be less common in SARS-CoV-2 ARDS than in “classic” ARDS and that prone positioning would result in small improvements in oxygenation with deterioration in overall respiratory system compliance, as a consequence of failure of lung recruitment.

## Main text

### Materials and methods

#### Study setting and design

Our study is a prospective cohort study of the first 20 patients with SARS-CoV-2 ARDS who underwent prone positioning in the intensive care unit (ICU) of our tertiary referral hospital. Included patients were admitted between the 16th March, 2020 and the 8th of April, 2020. Ethical approval was obtained from the local institutional review board. We included patients > 18 years of age who had laboratory confirmed SARS-CoV-2 infection, were invasively ventilated in the ICU, met the Berlin criteria for the diagnosis of ARDS [4] and underwent prone positioning as part of their management. Consent or assent was obtained as appropriate in accordance with the relevant local regulatory frameworks and national legislation. SARS-CoV-2 infection was confirmed using reverse transcriptase polymerase chain reaction testing on respiratory samples. All patients included were studied at the first session of prone positioning. Patients were identified from a prospective record of patients undergoing prone positioning in critical care areas. All patients included were ventilated in a mandatory volume control mode using ramped descending inspiratory flow and a lung-protective mechanical ventilation protocol. Institutional policy was that positive end-expiratory pressure (PEEP) should be set according to the ARDSNet PEEP tables [9]. Patients were excluded if they were younger than 18 years of age or, if due to surge demand exceeding capacity to maintain an electronic healthcare record (EHR) for all patients, they were cared for in areas where paper records were maintained and routine electronic data were not recorded. We also excluded patients who declined consent or where we could not obtain assent from the next of kin.

#### Data collection

Observations were obtained from analysis of routine clinical data in the EHR. We collected baseline data including demographic data and severity of illness data (PaO_2_/FiO_2_ (PF) ratio, SOFA score). For each patient we determined serial observations of ventilator parameters, measurements of respiratory mechanics and gas exchange before, during and after the first period of prone positioning. Plateau pressures were obtained at end expiration during zero-flow conditions.

ICU free days and ventilator free days (VFDs) were also determined from the EHR. 28-day mortality was also recorded. Ventilator free days were defined as days following intubation that the patient was alive and not mechanically ventilated for the 28-day period following their initial intubation. ICU free days were defined as any day not spent in a critical care area within the 28 days following their initial intubation.

#### Electrical impedance tomography (EIT)

We performed Electrical impedance tomography (EIT) in a further 3 patients with SARS-CoV-2 ARDS, using a clinical device as part of routine care (PulmoVista 500, Draeger Medical, Luebeck, Germany). Briefly, this noninvasive technique utilizes an electrode belt containing 16 electrodes, placed around the thorax in the fifth intercostal space, and one reference electrode placed on the abdomen. It’s measurement principle has been described in detail elsewhere and involves the creation of two-dimensional transverse single-slice images based on changes in impedance distribution originating from ventilation [10]. EIT can be used to assess lung recruitment [11]. We compared regional impedance variations 1 h before and after each patient’s first treatment with prone positioning.

#### Statistical analysis

Descriptive analyses were expressed as median (interquartile range [IQR]) for continuous variables and as percentages for categorical variables. Comparative statistics used repeated measures two-way analysis of variance (ANOVA) and Mann–Whitney U test as appropriate. For repeated measures two-way ANOVA we excluded patients where routine data were missing for relevant observations. All statistical analysis was performed using GraphPad version 8.0 (GraphPad Software, San Diego, USA).

### Results

During the study period 21 patients underwent prone positioning in the ICU. In total, 20 patients met the inclusion criteria and were included in the analysis. A single patient was treated in an area without an electronic health record system and thus was excluded from the analysis. The baseline characteristics of the final cohort (n = 20) are summarized in Table 1.

**Table 1:** Patient Characteristics, Blood Gas and Ventilatory Variables

Patient characteristics	Median (IQR)	
Age (years)	54.0 (45.0–59.5)	
Male (%)	90%	
BMI (kg/m^2^)	36.0 (30.0–43.4)	
SOFA score	8.0 (6.0–10.7)	
Duration between onset of symptoms and admission to ICU (days)	10.5 (7.2–15.0)	
Respiratory support prior to admission (NIV/HFNC), No. (%)	12 (60%) / 2 (10%)	
Duration of first prone positioning session, hours	16.2 h (15.6–17.4)	
Length of ICU stay prior to prone positioning	1 day (1–1.75)	
Arterial blood gas variables	Pre-prone positioning	During prone positioning
pH	7.30 (7.23–7.35	7.30 (7.22–7.36)
PaO_2_ (kPa)	12.5 (10.1–13.2)	14.3 (12.7–20.4)
PaCO_2_ (kPa)	7.0 (6.1–8.0)	7.3 (6.6–8.5)
Ventilatory variables		
Plateau airway pressure (cmH_2_O)	26 (20–28)	26 (22–29)
Tidal volume (mL)	426 (391–461)	436 (393–470)
PEEP (cmH_2_O)	14 (10–16)	14 (10–15)
FiO_2_ (%)	70 (60–95)	45 (36–55)
PaO2/FiO2 (mmHg)	123 (100–154)	286 (195–348)
Aa Gradient (mmHg)	342 (275–507)	114 (64–207)
C_RS_ (ml/cmH2O)	33.7 (30.1–43.0)	32.5 (26.7–37.5)

*BMI* body mass index, *SOFA* sequential organ failure assessment, *NIV* non-invasive ventilation, *HFNO* high flow nasal oxygen, *PaO*_*2*_ arterial partial pressure of oxygen, *PaCO*_*2*_ arterial partial pressure of carbon dioxide, *PEEP* positive end-expiratory pressure, *FiO*_*2*_ fraction of inspired oxygen, *Aa* alveolar-arterial, *C*_*RS*_ static compliance

The majority of patients were male, obese, with high severity of illness scores and had undergone a trial of either non-invasive ventilation or high flow nasal oxygen therapy prior to intubation. Most patients had moderate to severe ARDS by Berlin criteria. Low respiratory system static compliance (C_RS_) was common prior to prone positioning. All patients received low tidal volume ventilation (tidal volumes < 8 ml/kg predicted body weight) and the majority of patients spent at least 16 h in the prone position.

**Fig. 1:**
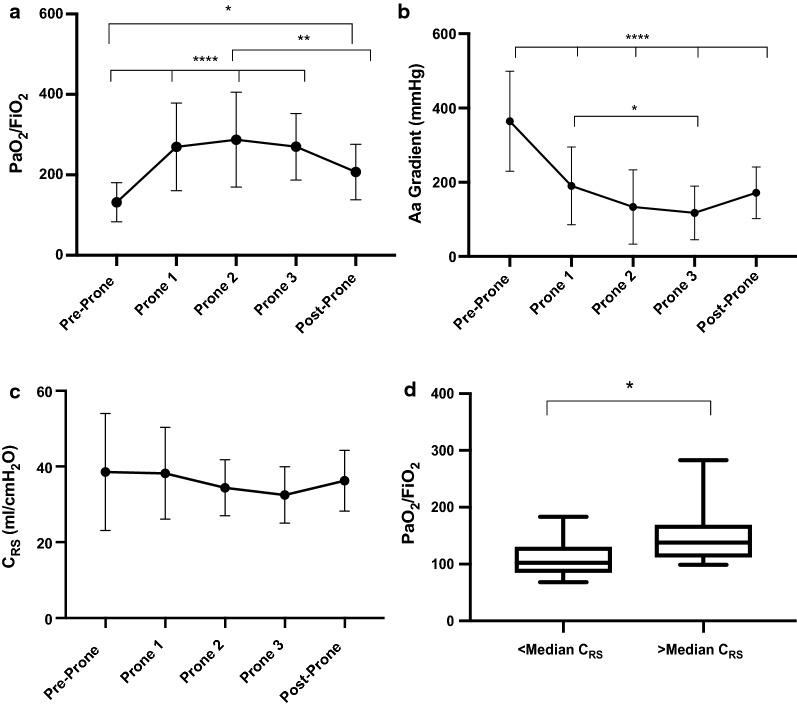
The effect of prone positioning on gas exchange and respiratory mechanics are shown in (**a–c**). **a ** Line graph representing mean PaO2/FiO2 ratio before, during, and after prone positioning, n=20, **b** Line graph representing mean Aa gradient before, during, and after prone positioning, n = 20, (**c**) Line graph representing mean respiratory system static compliance (CRS) before, during, and after prone positioning, n = 15, (**d**) shows the association between respiratory system static compliance (CRS) and severity of SARS-CoV-2 ARDS. It displays a box plot representing the difference in baseline PF ratio between patients with <median CRS and > median CRS, n = 19. PF = PaO2/FiO2 ratio, Aa = Alveolar-arterial gradient, CRS = respiratory system static compliance. Pre-Prone = immediately prior to prone positioning, Prone 1 = following prone positioning, Prone 2 = the mid-point of prone positioning, Prone 3 = prior to supination, and Post-Prone = following supination. Statistical Analysis: Analyzed by repeated measures two-way ANOVA with Tukey’s post-hoc test for multiple comparisons for line graphs and Mann-Whitney U test for box plot. ****P<0.0001, ** P<0.01, * P<0.05. Patients with incomplete data sets were excluded from analysis.** a–c**: error bars represent standard deviation.** d**: box plot with bars representing range.

The trend in PaO_2_/FiO_2_ ratios in the cohort before, during and after prone positioning is illustrated in Fig. 1a. There was a median improvement in the PaO_2_/FiO_2_ ratio of 132 in the prone position compared to the supine position (IQR, 67–228).The majority (90%) of patients experienced an increase in PaO_2_/FiO_2_ ratio of > 20% of baseline. Similarly, there was a significant and sustained decrease in Alveolar–arterial (Aa) oxygen gradient observed over the duration of prone positioning (Fig. 1b). The median decrease in Aa gradient was 212 mmHg (IQR, 134–359). There was no significant difference in C_RS_ noted throughout prone positioning (Fig. 1c). Patients with low (< median) C_RS_ had significantly lower baseline PF ratios when compared to those with higher (> median) C_RS_ (*P* < 0.05) (Fig. 1d).

**Fig. 2:**
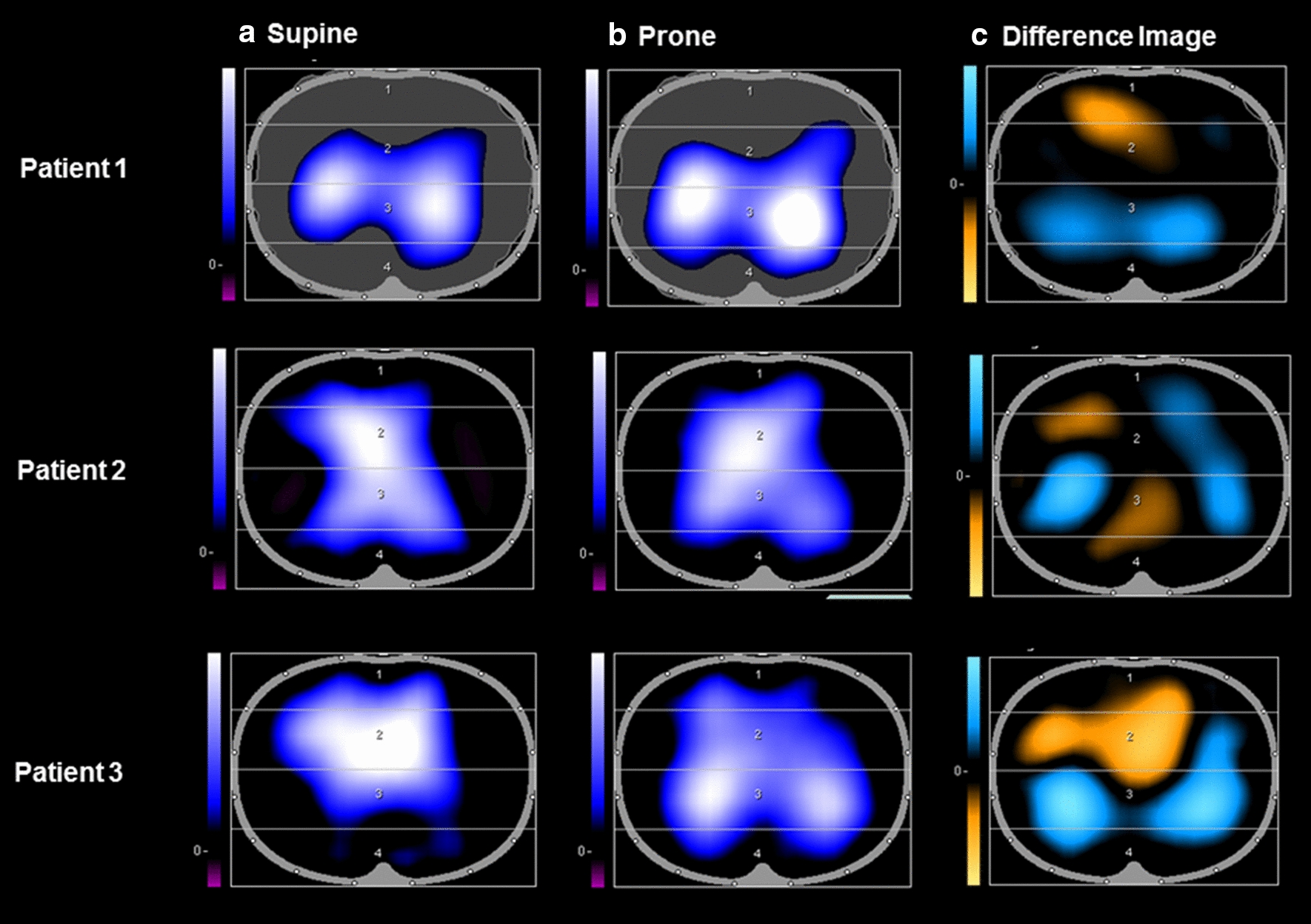
Electrical impedance tomographs (PulmoVista 500, Dräger) are shown above for 3 adult patients with SARS-CoV-2 ARDS who were invasively ventilated and underwent prone positioning.** a** represents the end-inspiratory trend view prior to prone positioning. ** b** represents the end-inspiratory trend view following prone positioning. In (**a, b**), areas of increasing impedance variation (corresponding to greater ventilation) are represented in order of increasing variation in black (none), blue (intermediate) and white (greatest) colors. ** c** represents the difference between the images in **a, b**, displaying loss of regional ventilation (areas in orange) which represent ventral regions being over-distended in the supine position and gain of regional ventilation (areas in blue) which represent recruitment of the dorsal regions upon prone positioning. Patients 1 and 3 showed an increase in tidal impedance variation in dorsal regions in the prone position and a decrease in tidal impedance variation in the ventral regions, which is consistent with lung recruitment dorsally.

The supine and prone comparisons of EIT measures of ventilation are presented in Fig. 2. Two out of three of the patients had evidence of early recruitment (increase in tidal impedance variation) in dorsal lung regions in the prone position compared to the supine position.

The majority of patients (85%) underwent further periods of prone positioning. A 28-day mortality rate of 15% was observed and the median number of ventilator free days among the cohort at 28 days was 16 (IQR, 0–21). The median number of ICU free days at 28 days in the cohort was 14.5 (IQR, 0–20).

### Discussion

In this prospective cohort study of invasively ventilated SARS-CoV-2 ARDS patients, we identified a marked and sustained improvement in measures of oxygenation in consecutive patients undergoing prone positioning. This improvement in gas exchange with prone positioning was not associated with a change in respiratory system static compliance.

We do not believe that our observations are consistent with SARS-CoV-2 ARDS representing an entity distinct from “classic” ARDS. Firstly, the vast majority (90%) of patients experienced an increase in PF ratio > 20% of baseline, which is consistent with previous observations in “classic ARDS” [12]. While the magnitude of this effect might appear greater than previously observed in “classic” ARDS [13–17], this is likely due to our early prone positioning strategy, which has previously been shown to be associated with improved oxygenation response [13]. Indeed a recent report of very early prone positioning in SARS-CoV-2 ARDS also observed an increased magnitude of effect [18].

Secondly, poor compliance was implicated in disease severity, and there was evidence of lung recruitability, both of which are characteristics of “classic” ARDS. In the first instance there was a strong association between more severe SARS-CoV-2 ARDS and poorer static compliance in our cohort. Patients with lower static compliance had lower baseline PF ratios. Absolute levels of compliance were low and comparable with previous studies in “classic” ARDS [19, 20]. Also, we did not observe a reduction in static compliance during prone positioning, as would be expected if lung recruitment did not occur. As we know that chest wall compliance consistently falls during prone positioning [8], this must mean that lung compliance improved (because total respiratory compliance is the sum of chest wall compliance and lung compliance). This appears most likely to be due to recruitment of poorly compliant lung in the prone position, as occurs in “classic” ARDS. While total compliance did not improve, this is actually a very common finding in prone positioning in “classic” ARDS [8]. Moreover, serial electrical impedance tomography (EIT) measurements in a small convenience sample demonstrated recruitment of dorsal lung regions in the prone position in two of three patients.

Our observations may conflict with previous data indicating that the majority of patients with SARS-CoV-2 ARDS have relatively normal lung compliance [5, 21] but agree with a more recent dataset [18]. The response to prone positioning in our cohort seems typical of “classic” ARDS. It could be argued that this provides grounds to generalise the findings of improved mortality with prone positioning in “classic” ARDS to patients with SARS-CoV-2 ARDS. However, randomized controlled trials would be needed to definitively confirm this.

### Conclusion

Prone positioning was effective in improving oxygenation in SARS-CoV-2 ARDS. Furthermore, poor respiratory system static compliance was common and improvements in oxygenation were partly due to recruitment of poorly compliant lung. Prone positioning should be considered in patients with SARS-CoV-2 ARDS.

### Limitations

Our study had several limitations. Firstly, the small convenience sample and the single-centre, observational nature of the study may limit generalisability. We used routine data and our conclusions about lung compliance are based on inferences based on total respiratory system compliance rather than direct measurements of lung compliance. Additionally, while selection bias could have influenced patient characteristics observed, we do not believe that this is a significant issue as the vast majority (73%) of COVID-19 ARDS patients admitted to our ICU during the study period underwent prone positioning.

## Data Availability

The datasets generated and/or analysed during the current study are not publicly available due to relevant data protection laws but may be available from the corresponding author on reasonable request.

## Abbreviations

ARDS: Acute respiratory distress syndrome
COVID-19: The disease caused by SARS CoV-2 infection
C_RS_: Total respiratory system static compliance
EHR: Electronic health record
EIT: Electrical impedance tomography
ICU: Intensive care unit
PEEP: Positive end expiratory pressure
PF Ratio: PaO2/FiO2 ratio
SARS CoV-2: Severe acute respiratory syndrome coronavirus-2
SOFA: Sequential organ failure assessment
VFD: Ventilator free days
